# At-Home Virtual Reality Intervention for Patients With Chronic Musculoskeletal Pain: Single-Case Experimental Design Study

**DOI:** 10.2196/58784

**Published:** 2025-03-04

**Authors:** Syl Slatman, Lieke Heesink, Reinoud Achterkamp, José Broeks, Nelson Monteiro de Oliveira, Remko ter Riet, Marjolein Stegeman, Monique Tabak

**Affiliations:** 1 Biomedical Signals and Systems Group, Faculty of Electrical Engineering, Mathematics and Computer Science, University of Twente, Drienerlolaan 5, Enschede, 7522NB, Netherlands; 2 Musculoskeletal Rehabilitation Research Group, School for Allied Health, HAN University of Applied Sciences, Nijmegen, Netherlands; 3 Health Technology and Services Research, Faculty of Behavioural, Management and Social Sciences, University of Twente, Hallenweg 5, Enschede, 7522NH, Netherlands, 31 534899128; 4 Department of Chronic Pain, Roessingh Rehabilitation Center, Enschede, Netherlands; 5 Nocepta, Hengelo, Netherlands

**Keywords:** virtual reality, VR, chronic musculoskeletal pain, CMP, single-case experimental design, SCED, user experience, self-management, musculoskeletal pain

## Abstract

**Background:**

Virtual reality (VR) could possibly alleviate complaints related to chronic musculoskeletal pain (CMP); however, little is known about how it affects pain-related variables on an individual level and how patients experience this intervention.

**Objective:**

This study aimed to gain detailed insight into the influence of an at-home VR intervention for pain education and management on pain-related variables, and to explore its feasibility and general experience.

**Methods:**

The study applied a single-case experimental design in which an at-home VR intervention was used for 4 weeks by patients with CMP who were on a waiting list for regular pain treatment. Outcome measures included pain-related variables, functioning, and objectively measured outcomes (ie, stress, sleep, and steps). Outcomes were analyzed using data visualization (based on line plots) and statistical methods (ie, Tau-U and reliable change index) on an individual and group level. In addition, a focus group was conducted to assess feasibility and general experience to substantiate findings from the single-case experimental design study. This focus group was analyzed using inductive thematic analysis.

**Results:**

A total of 7 participants (female: n=6, 86%) with a median age of 45 (range 31‐61) years participated in this study. A dataset with 42 measurement moments was collected with a median of 280 (range 241‐315) data points per participant. No statistically significant or clinically relevant differences between the intervention and no-intervention phases were found. Results of the visual analysis of the diary data showed that patients responded differently to the intervention. Results of the focus group with 3 participants showed that the VR intervention was perceived as a feasible and valued additional intervention.

**Conclusions:**

Although patients expressed a positive perspective on this VR intervention, it did not seem to influence pain-related outcomes. Individual patients responded differently to the intervention, which implies that this intervention might not be suitable for all patients. Future studies should examine which CMP patients VR is effective for and explore its working mechanisms. In addition, future larger trials should be conducted to complement this study’s findings on the effectiveness of this intervention for patients with CMP and whether VR prevents deterioration on the waiting list compared with a control group.

## Introduction

Chronic musculoskeletal pain (CMP), defined as pain lasting longer than 3 months, is a major problem and prevalent in approximately 20% of adults [1,2]. CMP is associated with a decrease in quality of life and mental health problems [3,4], next to the significant financial and societal burden [1]. Unfortunately, the effectiveness of biomedical treatment options for CMP does not seem to be very promising [5], since CMP usually is a complex problem with an interplay of biological, psychological, and social factors [6].

Given the complexity of CMP, treatment should use a holistic approach in accordance with the biopsychosocial model [5] and neuromatrix theory [7]. Unfortunately, most more complex, holistic interventions for CMP have a waiting list period, which could have a deteriorating effect on patients with CMP [8]. Therefore, it might be sensible to already start treatment during this waiting list period. Virtual reality (VR) is a novel, therapeutic technology that is suitable for stand-alone, at-home treatment [9]. VR is defined as “a collection of technologies that allow people to interact efficiently with 3D computerized databases in real time using their natural senses and skills” [10].

Even though VR for CMP seems promising, much is still unknown about its underlying mechanisms (eg, distraction or skills-building) [11] and influences on an individual level, as previous studies applied a nomothetic approach [9]. Since the principles underlying VR for CMP remain a black box [12], an idiographic approach is warranted for a complex condition like CMP to gain insight into the influence of VR on individual outcomes [13]. A single-case experimental design (SCED) study could increase understanding of the individual experience [14]. SCED studies apply detailed assessment at numerous timepoints [15] and have benefits over other designs, including patients serving as their own control and being especially suitable for heterogeneous samples, like CMP patients with a variety of conditions [16]. A recent SCED study on VR for chronic low back pain (CLBP) found that VR has the potential to reduce CMP-related complaints, possibly through a combination of distraction and modification of attitudes and beliefs [17]. We expect that this VR intervention is suitable not only for patients with CLBP but also for patients with other CMP conditions. In addition, we hypothesize that VR might influence other outcome measures like pain acceptance and interference, functioning, and objectively measured outcomes.

Therefore, the aim of our study was to (1) explore whether and how a VR intervention has an influence on pain-related variables on an individual level and (2) explore the feasibility and general experience of the VR intervention. To do so, patients with CMP received a pain education and management VR intervention at home while they were on a waiting list to receive pain treatment.

## Methods

### Design

This mixed methods study consisted of 2 parts. The first part of the study applied a nonconcurrent single-case experimental ABA-design on at-home, VR intervention for patients with primary or secondary CMP who were on a waiting list to receive regular pain treatment. Phases A1 and A2 (no intervention) were 1 week before and 1 week after the VR intervention, fulfilling the criterion for a sufficient baseline in single-case designs [18]. Phase B (VR intervention) lasted a total of 4 weeks. To report and conduct the study, the Single-Case Reporting Guideline in Behavioural Interventions (SCRIBE) was used [19], more details in Multimedia Appendix 1. The second part of this study consisted of 1 focus group with patients with CMP who received the intervention. The aim of this focus group was to gain more insight into the general experience and feasibility (including acceptability and practicality, which includes participants’ satisfaction and ability to use a new intervention [20]) of the VR intervention and substantiate findings from the SCED study. This part of the study was reported and conducted according to the Consolidated Criteria for Reporting Qualitative Research (COREQ) reporting guidelines [21], more details in Multimedia Appendix 2. Recruitment and completion of the study procedures was from February 2023 to April 2023.

### Ethical Considerations

The medical ethics committee of Radboudumc provided a non-WMO (medical research involving human subjects act) waiver (2022‐15829) to conduct this study. The ethics committee of the University of Twente approved this study (RP 2022‐174), as well as local ethics committees of the participating health care organizations. Participants gave written informed consent before any study procedures and received €50 (US $52) for participation in this study after finishing all procedures. All participant data was pseudonymized.

### Participants

Participants were recruited from 4 secondary care organizations in the Netherlands (ie, Roessingh Centrum voor Revalidatie, Roessingh Pijnrevalidatie, ZGT Nocepta, and Deventer hospital). Patients were deemed eligible for participation if they (1) were aged 18 years or older, (2) had primary or secondary CMP, (3) finished first-line treatment, (4) were open to treatment with biopsychosocial elements, and (5) were willing and able to comply with the study protocol. Patients were excluded if they (1) were not capable of finishing the intervention due to physical (eg, face wounds, severe visual impairment), mental (eg, severe sensitivity to stimuli), or practical problems (eg, insufficient tech literacy); and (2) had no comprehension of the Dutch language.

### Intervention

In this study, the Conformité Européenne (CE)–certified VR intervention Reducept was used as a daily at-home intervention for 10 to 30 minutes per day for 4 weeks, thereby following the intervention protocol dosage from the intervention provider. Besides pain neuroscience education (PNE), the VR intervention incorporates elements of several psychological therapies into 1 application: hypnotherapy, mindfulness, acceptance and commitment therapy (ACT), and cognitive behavioral therapy (CBT). The intervention was described in more detail in previous studies [9,22,23]. The Pico G2 4K (Bytedance) head-mounted display (HMD) was used in this study to provide the immersive VR intervention.

### Procedure

Patients visited one of the participating centers of this study for their pain treatment. After their intake, but before starting their secondary care treatment (either [non]invasive pain treatment or interdisciplinary pain rehabilitation), patients were screened by their health care professional for possible participation in the study. Patients were given the opportunity to participate in our study or wait for their treatment on the waiting list without receiving any other treatment. In addition, participants were made clear that participating in this study would not have any influence on the pain treatment they were on a waiting list for. If a patient was deemed eligible, he or she was contacted by their health care professional, who gave a brief explanation about the study and asked for permission to forward the patient’s contact details to the researcher (through a fully secured app: Siilo). Next, the researcher contacted the patient by phone and gave more detailed information about the study and asked the patient to contemplate participating in the study. The patient enrolled in the study by signing the informed consent and received the first questionnaires (T0), the Garmin Forerunner 255 wearable, and the VR headset. The wearable and VR headset were provided by the researcher and used by participants for the duration of the study procedures. In the first week, a detailed baseline was obtained by asking patients to use the wearable and fill in the diary and weekly questionnaires, without receiving the intervention (phase A1). After this phase, participants carried out the intervention at home for four weeks (phase B). Next, patients waited a week (phase A2) before receiving the pain treatment he or she was on the waiting list for. After phase A2 and during the period patients received the pain treatment they were on a waiting list for, patients returned the used equipment (ie, VR headset and wearable) and were invited to the online focus group, using Microsoft Teams, about the feasibility and general experience of the intervention. The focus group was conducted by 2 researchers (SS and LH), assisted by a research student assistant. Both SS and LH attended various courses on and have previous experience with qualitative research. Given this experience, there may have been preconceived notions regarding VR for CMP. We aimed to reduce potential biases by fostering open discussions and critical reflections throughout data collection and analysis. None of the participants had previous relationships with any of the researchers conducting and analyzing the focus group. The topic list used for this focus group is added in Multimedia Appendix 3.

### Outcomes

The outcome measures are shown in Table 1. The TIIM app (University of Twente, Enschede, the Netherlands) was used to collect demographic information, diary measures, and weekly questionnaires.

**Table 1.** Overview of outcome measurements.

	Pre	Week 1	Week 2	Week 3	Week 4	Week 5	Week 6	Post
Patient characteristics	✓							
Diary measures		✓	✓	✓	✓	✓	✓	
Weekly questionnaires		✓	✓	✓	✓	✓	✓	
Wearable data		✓	✓	✓	✓	✓	✓	
VR^a^ parameters			✓	✓	✓	✓		
Feasibility								✓

a VR: virtual reality.

### Diary Measures

The daily diary questions consisted of 4 questions, based on the IMMPACT (Initiative on Methods, Measurement, and Pain Assessment in Clinical Trials) recommendations for chronic pain clinical trials [24]: pain intensity (ie, what score would you give your pain today?), pain interference (ie, how burdensome was your pain today?), physical functioning (ie, to what extent did your pain restrict you in doing daily activities today?), and emotional functioning (ie, how was your mood today?). All questions were scored on a 0 (lowest) to 10 (highest) scale. A recent study showed that daily measures of pain and pain-related variables are both valid and reliable [25].

### Weekly Questionnaires

Every week, participants were asked to answer 3 questionnaires to measure pain self-efficacy (Pain Self-Efficacy Questionnaire [PSEQ]) [26], pain acceptance (Chronic Pain Acceptance Questionnaire [CPAQ]) [27], and pain coping (Pain Coping Inventory [PCI]) [28]. These questionnaires were the Dutch translation of the original questionnaires, and all were shown to have adequate reliability and validity [29-31].

### Wearable Outcomes

The following outcomes were measured using the wearable: physical activity (ie, daily steps), sleep quality, and stress. Daily sleep quality was scored from 0 (worst sleep quality) to 100 (best sleep quality) based on multiple factors, including sleep duration, stress score during sleep, and restlessness. Daily stress was measured using Garmin’s stress level from 0 (lowest stress level) to 100 (highest stress level), which is based on the participant’s heart rate variability (HRV). More information about the construction of sleep quality and stress as outcome measures in this study can be found in the Garmin manual [32].

### Other Outcomes

The following patient characteristics were asked at baseline: age, gender, duration of CMP, comorbidities, pain location, pain medication use, expectation of intervention, occupational situation, education level (based on [33]), and experience with VR for treatment and entertainment.

VR-related parameters that were monitored included usage and module of the VR intervention.

The feasibility of the intervention was explored using usability data (ie, number of minutes used per day) and a semistructured postintervention focus group with patients who received the intervention.

### Statistical Analysis

The results of the SCED study were examined using a combination of statistical and visual analyses [34,35]. Phase A1 of each individual participant was observed to determine a stable personal control to note any revealing alterations for the outcome variables measured in phase B. Both within-phase and between-phase analyses were performed and checked for patterns within participants. To determine changes in outcome variables in SCED studies, it is recommended to use the following factors to interpret the data: (1) raw data, (2) central tendency, (3) trend, (4) variability, (5) point of change, and (6) overlap region [15]. All visual plots were constructed using the Shiny SCDA web application [36,37]. Besides this visual analysis, outcomes of the diary questions and wearable data were statistically analyzed using the Tau-U nonoverlap method [38], using a web-based calculator [39]. Effect sizes for Tau-U were interpreted as small (0-.65), medium (.66-.92), or large (>.92) [38]. To gain insight into the relationship between pain-related variables during the intervention, outcomes of the weekly questionnaires were compared on an individual level using the Reliable Change Index (RCI). The RCI was calculated using the pretreatment and posttreatment scores and was considered reliable at 1.96 or more [40]. Clinically important differences in pain intensity were examined between pre- and postintervention, in which a reduction of ≥30% or 2 points was considered clinically important [41]. The recording of the focus group, which had a duration of 50 minutes, was transcribed using Amberscript. This transcript was analyzed using inductive thematic analysis with Atlas.ti (version 24), based on the 6 steps proposed by Braun and Clarke [42]: (1) (re-)read transcript to familiarize with the data, (2) generate initial codes, (3) combine codes into themes, (4) review themes, (5) define themes, and (6) report findings. These steps were completed by 2 researchers (SS and LH) and discussed until consensus was reached. Finally, all authors agreed on the final themes and results identified during this process.

## Results

### Patient Characteristics

A total of 9 participants enrolled in this study, of which 7 completed the study (Table 2). In addition, 1 participant stopped due to being too busy and 1 participant completed <50% of the questionnaires and was therefore excluded from the analysis. The 7 participants who were included in the analysis provided a median of 280 (range 241‐315) data points per participant. None of the participants had previous experience with VR. No adverse events were reported by any of the participants from using the VR intervention.

**Table 2.** Demographics of participants (n=7).

Participant	Age (years)	Gender	Highest level of education	Occupational situation	Pain duration (years)	Pain location	Medication use	Expectancy^a^
1	31	Woman	Higher	Part-time	1	Foot, ankle	Yes	6
2	55	Man	Lower	Full-time	17	Legs, hands	Yes	5
3	45	Woman	Middle	Part-time	5	Wrist, shoulder, back	Yes	4
4	31	Woman	Middle	Unemployed	7	Generalized	No	6
5	61	Woman	Lower	Part-time	30	Back, hip	Yes	6
6	52	Woman	Higher	Full-time	3	Back, shoulders, neck	Yes	5
7	37	Woman	Higher	Part-time	4.5	Back, pelvic	Yes	6

a Scored from 0 (lowest expectancy) to 10 (highest expectancy).

### Visual Analysis

**Figure 1.**
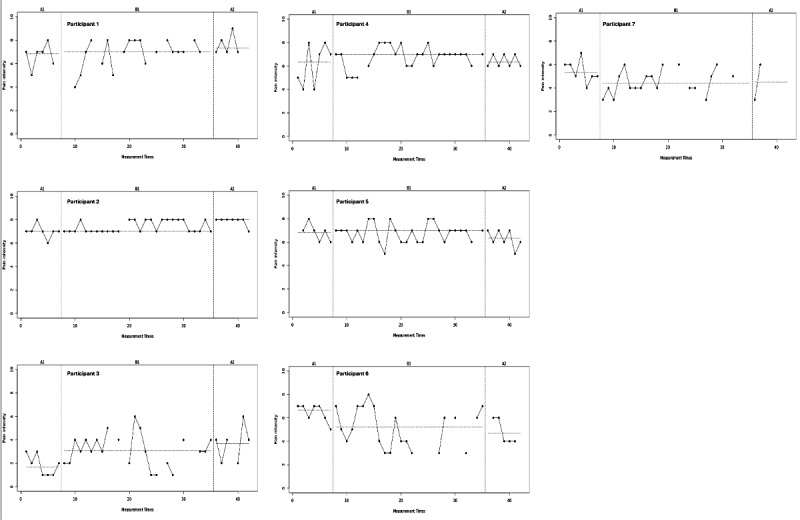
Visual analysis of diary data on pain intensity (see clearer version in Multimedia Appendix 5).

Results of the visual analysis of the diary data showed that patients responded differently to the intervention, as discussed below per outcome variable. The results of the 4 diary outcome measures are presented in Figures1  2 and Multimedia Appendix 4, in which the phases A1 (day 1‐7, no intervention), B (day 8‐35, intervention), and A2 (day 36‐42, no intervention) are presented on the x-axis and scores from 0 (lowest) to 10 (highest) are presented on the y-axis.

**Figure 2.**
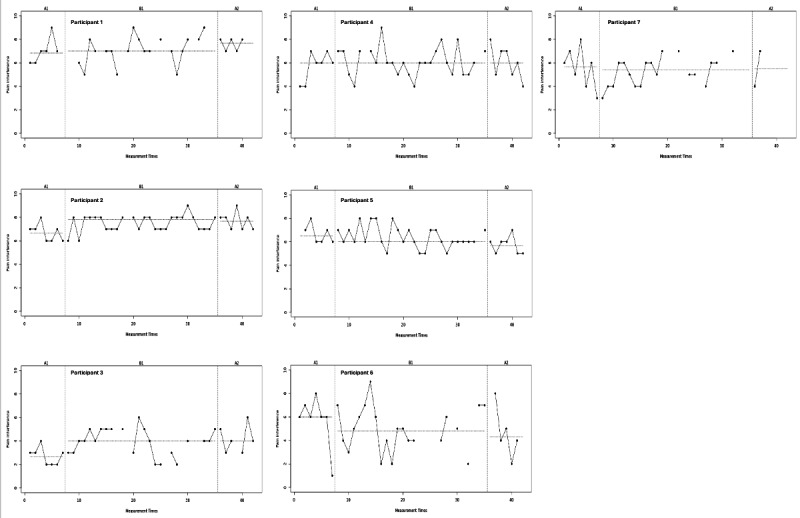
Visual analysis of diary data on pain interference (see clearer version in Multimedia Appendix 6).

Pain intensity scores (Figure 1) remained relatively consistent through phase A1, B, and A2. However, some participants seem to report somewhat lower scores during phase B compared with phase A1 (eg, participant 6 from mean phase A1 6.4, SD 0.8, to mean phase B 5.1, SD 1.7), while others report higher scores (eg, participant 3 from mean phase A1 1.9, SD 0.9 to mean phase B 3.3, SD 1.4). Furthermore, it is notable that most participants reported substantial variability within proximate measurement moments.

Analysis of the pain interference outcome (Figure 2) showed that patients reported fairly stable scores on central tendency. Some participants showed minor improvement between phases (eg, participant 2 from mean phase A1 6.7, SD 0.8, to mean phase B 7.5, SD 0.7), while others showed some deterioration (eg, participant 5 from mean phase B 6.4, SD 0.9, to mean phase A2 5.7, SD 0.8). In addition, it should be noted that pain interference scores show much likeness to pain intensity scores.

Results on physical functioning (Multimedia Appendix 4) showed that central tendency does not seem to alter too much between phases, similar to the results on pain intensity and pain interference scores. Variability within patients seems to be similar to previously reported outcome measures as well, except for participant 3 who shows large variability within proximate measurement times (eg, day 23: 2; day 24: 10; day 25: 2).

Finally, emotional functioning scores (Multimedia Appendix 4) were relatively high in most participants (mean 7.1, SD 1.5, compared with mean pain intensity 5.9, SD 1.8, pain interference 5.9, SD 1.8, and physical functioning 5.4, SD 1.7). Trend between phases seemed to be improving for some participants (eg, phase A1 of participant 7), while the opposite occurred in other participants (eg, phase A2 of participant 4). Variability seemed to be lower compared with previously discussed outcome measures in most participants.

### Statistical Analysis

Analysis of the daily diary and wearable data using Tau-U, as shown in Table 3, showed no statistically significant difference in any of the outcome measures. In addition, no clinically important reductions in pain intensity (ie, reduction of pain intensity score of ≥30% or ≥2 points) were found. Results of the statistical analysis of the weekly questionnaires using the RCI (Table 4) showed no reliable change on any of the questionnaires for any of the participants. More detailed information about the results of the wearable data and weekly questionnaires can be found in respectively Multimedia Appendix 7 (individual scores on steps, stress, and sleep) and Multimedia Appendix 8 (Group scores on weekly questionnaires). Median VR use was 37.5 minutes per week (range 7.8‐78.4).

**Table 3.** Statistical analysis of diary and wearable data.

	Tau-U	95% CI	*P* value
Pain intensity	−0.011	−0.16 to 0.14	.88
Pain interference	−0.013	−0.16 to 0.13	.87
Physical functioning	−0.091	−0.24 to 0.06	.23
Emotional functioning	−0.021	−0.17 to 0.13	.78
Steps	0.013	−0.14 to 0.17	.87
Stress	−0.075	−0.23 to 0.09	.36
Sleep	0.082	−0.08 to 0.24	.32

**Table 4.** Statistical analysis of weekly questionnaires.

	Participant						
	1	2	3	4	5	6	7
PSEQ^a^							
Pretreatment, mean (SD)	43 (0.7)	31 (3.5)	42 (4.2)	21 (8.5)	37 (4.9)	23 (2.8)	27 (0)
Posttreatment, mean (SD)	38 (2.8)	36 (0)	47 (2.1)	23 (2.1)	45 (1.4)	18 (2.1)	29 (3.5)
RCI^b^	−1.05	1.05	1.05	0.42	1.68	−1.05	0.42
CPAQ^c^							
Pretreatment, mean (SD)	23 (0)	32 (0.7)	31 (0.7)	20 (0.7)	29 (1.4)	15 (1.4)	18 (5.7)
Posttreatment, mean (SD)	28 (1.4)	31 (3.5)	31 (2.8)	23 (0)	29 (1.4)	15 (2.1)	20 (2.1)
RCI	0.74	−0.15	0	0.45	0	0	0.30
PCI^d^ active							
Pretreatment, mean (SD)	31 (0.7)	31 (1.4)	31 (1.4)	29 (0.7)	26 (0.7)	28 (0.7)	30 (1.4)
Posttreatment, mean (SD)	28 (1.4)	28 (0)	34 (0)	26 (0)	27 (2.8)	23 (1.4)	30 (0.7)
RCI	−0.84	−0.84	0.84	−0.84	0.28	−1.40	0
PCI passive							
Pretreatment, mean (SD)	40 (1.4)	44 (5.7)	42 (0)	64 (2.8)	46 (3.5)	49 (0.7)	51 (4.2)
Posttreatment, mean (SD)	43 (4.2)	44 (0.7)	36 (.7)	59 (1.4)	44 (0.7)	45 (0)	55 (1.4)
RCI	−0.38	0	0.77	0.64	0.26	0.51	−0.51

a PSEQ: Pain Self-Efficacy Questionnaire.

b RCI: Reliable Change Index.

c CPAQ: Chronic Pain Acceptance Questionnaire.

d PCI: Pain Coping Inventory.

### Focus Group Analysis

Participants 4, 6, and 7, as described in Table 2, participated in the postintervention focus group. The other participants were not able to participate because they were too busy (with their pain rehabilitation program) (n=3), and did not feel well on the day of the focus group (n=1). Based on the analysis of the focus group, the following three themes were identified: (1) experiences of CMP patients with VR, (2) feasibility of VR, and (3) VR in CMP rehabilitation.

#### Theme 1: Experiences of CMP Patients With VR

Participants found the VR program attractive to use and valued the intuitive nature of the intervention. Furthermore, they reported several positive effects of the VR intervention, including feelings of self-efficacy, more knowledge about (chronic) pain and focus shifting. Although, these effects were not substantial and patients had to get used to using VR, as it demanded both their time and effort.

*And it provided me with insights about how chronic pain works.*

[Participant 7]

*My focus shifted away from the pain and went more towards the game or killing those monsters, which was a lot of fun. And then you notice that it does something with the pain.*

[Participant 6]

*And then you still [use VR] while you are actually already tired and in need of a bit of a rest.*

[Participant 4]

#### Theme 2: Feasibility of VR

Participants perceived the VR intervention as feasible. They found it easy and comfortable to use at home, the instructions were clear, and it was attainable to use daily.

*And we received clear instructions beforehand, so then it’s just plug and play, you know.*

[Participant 4]

*Yes, I think I actually liked using it at home first, instead of somewhere else.*

[Participant 6]

#### Theme 3: VR in CMP Rehabilitation

VR helped participants bridge the waiting time, but participants valued it more as an addition to their treatment rather than a substitution.

*It’s more of an addition, a good addition, a meaningful addition*.

[Participant 6]

Some participants mentioned it might be valuable to provide the VR intervention not only during the waiting list period but also during the pain treatment they were on the waiting list for. Furthermore, it is important to consider the individual process and whether a patient is open to working on the topics addressed in the VR intervention.

*…that it would be even more effective during pain treatment, it would be even stronger, because you are already more involved in it and you can also ask for feedback immediately, for example from one of your therapists, if you have any questions.*

[Participant 7]

*It [the VR intervention] raised some internal conflict, but I can really understand that it could be very helpful for patients who are further in their process.*

[Participant 4]

In the future, patients would recommend to receive VR not on a daily basis, but maybe 2 or 3 times a week, in between the days of the pain rehabilitation program.

## Discussion

### Principal Findings

The aim of this study was to gain insight into the influence of VR on pain-related variables and evaluate the feasibility and general experience of this intervention. Analyses of the reported measures showed no clinical and statistically significant differences. Our results imply that the provided intervention did not influence the outcome measures used in this study. This was supported by the visual analyses, which showed that some participants somewhat improved after the intervention on several outcome measures, but worsened on different outcome measures. However, results of the focus group showed that patients qualitatively reported a positive perspective and experienced the intervention as feasible.

### Comparison to Previous Work

The results of this study are comparable to other studies that provided the VR intervention, Reducept. A previous study that examined the effect of Reducept for patients with CLBP who were on a waiting list to receive pain treatment [9], showed no significant between-group results on the primary and most other outcome measures, except for opioid use, daily worst, and least experienced pain intensity. It should be noted that the patient sample in both their and our study were patients with severe and complex symptoms. They were referred to secondary pain care, with for example a median pain duration of 5 years in our sample. Previous studies showed that a longer duration of pain complaints was associated with a worse prognosis [43,44] and diminished responsivity to treatment [45]. As suggested before, this specific stand-alone VR intervention might therefore be more suitable for CMP patients with less complex complaints [17].

This study by de Vries et al [17] found somewhat more promising results when they conducted a SCED study among patients with CLBP where they received 9 to 12 45-minute sessions of the VR intervention [17]. Results of their study showed that Reducept might be able to induce clinically relevant reductions in pain intensity and other pain-related outcomes in some patients [17]. These patients were not on a waiting list to receive other pain treatment and received the intervention supervised in the hospital, which might have increased effectiveness [46]. Other interventions that used a stand-alone at-home VR intervention reported clinically meaningful results [47-49], but patients were (1) not on a waiting list to receive other pain treatment and (2) received a more extensive intervention (both in duration and content). A waiting list period is known to possibly deteriorate pain complaints [8]. A meta-analysis among psychotherapies even showed that waiting lists might be regarded as a nocebo condition since patients might, for example, feel the need to remain their complaints to be able to start the pain treatment they are on the waiting list for [50]. In addition, it might be possible that the waiting list period is not the best time to provide VR. This was mentioned in our focus group, and previous research showed that it is also possible to extend secondary care for CMP patients with VR as an additional treatment option [51,52]. In regard to the content of the VR module, it might be possibile to supplement this with, for example, personalized exercise therapy as was done in previous VR interventions for CMP [51,53,54]. Finally, the dosage of the VR intervention might be a point of interest, as the study by de Vries et al [17] found different results from this study while using another dosage of the same intervention. The intervention duration in this trial was 4 weeks, while for behavioral CMP interventions, a duration of 6 to 10 weeks is advised [55], which implies that the intervention did not last long enough. Future studies on VR for CMP should, therefore, study the optimal timing, (personalized) content, and dosage of VR interventions for the most fitting patients.

Results of our study showed a discrepancy between the analyses of quantitative outcome measures and qualitative measures. This is congruent with the qualitative evaluation [22] of the trial that was discussed before [9]. They reported that the VR intervention positively affected how patients’ health was experienced, provided patients with more control over their pain, and helped patients accept and understand pain. This is supported by other studies in which patients did not report significant differences in, for example, quality of life or pain intensity measured using questionnaires but mentioned positive benefits during an oral evaluation after their VR intervention [17,56]. This discrepancy could partially be explained by social-desirability bias, as patients might want to portray a more positive impression of the intervention for the researcher who is interviewing them [57]. In addition, it might be possible that nonoptimal quantitative outcome measures were used for this VR intervention, and softer outcomes like values (eg, autonomy) or more proximate outcomes (eg, knowledge about CMP) should be examined as well, as was suggested previously [14].

### Strengths and Limitations

One of the strengths of this study was the use of a heterogeneous sample of patients with ranging ages (31-61 years), pain duration (1-30 years), and type of pain complaints. In addition, a rich dataset with multiple subjective (ie, daily diary, validated questionnaires, and focus group) and objective (ie, wearable) outcome measures was used, which was analyzed both visually and statistically. In line with SCED study recommendations, at least 5 data points per phase were collected [58].

This study had several limitations. First, the nature of the study design is characterized by a smaller sample size, which came with risks of selection-bias of specific patients and hindered generalizability of study results. Second, treatment fidelity varied between participants, and not all participants used the VR intervention as much as prescribed, which could have diminished the intervention effect. This problem was mentioned in other VR interventions for CMP as well [48,53], while it is known that repetition is key in, for example, PNE [59]. However, it should be noted that treatment fidelity varies outside a study design, and therefore, this study reflects a real-world situation. Third, we conducted only 1 focus group with 3 participants who provided an insight into the intervention feasibility. Given the limited sample size, these results should be interpreted with caution. However, a more in-depth analysis of qualitative data, possibly with one-on-one interviews instead of focus groups, of participants’ experience with VR in a larger study sample would be interesting, to learn more about possible working mechanisms and administration best practices of VR for CMP, which could further improve this intervention.

### Future Directions

The results of this study suggest implications for clinical and theoretical practice. It seems that this stand-alone VR intervention for patients with CMP on a waiting list for secondary care does not influence pain-related complaints. However, in the right dose, setting, and timing it might be more effective, as previous research, for example, suggested that VR interventions for CMP might be more effective for younger patients [60]. To further inform trial and intervention design, other relevant pain-related outcomes (eg, catastrophizing) and medication use could be investigated, as these were found relevant in previous VR for CMP studies [9]. In addition, future studies could explore prognostic patient characteristics to identify patients who would respond better or worse to therapeutic VR for CMP. To further study the effectiveness of the (improved) intervention and complement the findings of this study, a randomized controlled trial (RCT) is warranted, in which a control group that receives usual care should be included. This RCT should both focus on the short-term results and include an analysis of the complete pain treatment trajectory. Furthermore, subgroup analyses are needed to examine for which patients VR is effective.

The results of this study showed that this stand-alone immersive VR intervention for patients with CMP on a waiting list did not seem to alter pain-related outcomes. Patients reported good feasibility and general positive experience of the intervention and these outcomes can inform further intervention and trial design.

## Supplementary material

10.2196/58784 Multimedia Appendix 1 Single-Case Reporting Guideline in Behavioural Interventions (SCRIBE) checklist.

10.2196/58784 Multimedia Appendix 2 Consolidated Criteria for Reporting Qualitative Research (COREQ) checklist.

10.2196/58784 Multimedia Appendix 3 Topic list focus group.

10.2196/58784 Multimedia Appendix 4 Visual analysis of diary data on physical and emotional functioning.

10.2196/58784 Multimedia Appendix 5 Clearer version of “Visual analysis of diary data on pain intensity.”

10.2196/58784 Multimedia Appendix 6 Clearer version of “Visual analysis of diary data on pain interference.”

10.2196/58784 Multimedia Appendix 7 Individual scores on steps, stress, and sleep.

10.2196/58784 Multimedia Appendix 8 Group scores on weekly questionnaires.

## Abbreviations

ACT: acceptance and commitment therapy
CBT: cognitive behavioral therapy
CE: Conformité Européenne
CLBP: chronic low back pain
CMP: chronic musculoskeletal pain
COREQ: Consolidated Criteria for Reporting Qualitative Research
COREQ: Consolidated Criteria for Reporting Qualitative Research
CPAQ: Chronic Pain Acceptance Questionnaire
HMD: head-mounted display
HRV: heart rate variability
IMMPACT: Initiative on Methods, Measurement, and Pain Assessment in Clinical Trials
PCI: Pain Coping Inventory
PNE: pain neuroscience education
PSEQ: Pain Self-Efficacy Questionnaire
RCI: Reliable Change Index
RCT: randomized controlled trial
SCED: single-case experimental design
SCRIBE: Single-Case Reporting Guideline in Behavioural Interventions
VR: virtual reality
