# Supplementary material for: At-Home Virtual Reality Intervention for Patients With Chronic Musculoskeletal Pain: Single-Case Experimental Design Study
Source: JMIR XR Spat Comput. 2025 Mar 4;2:e58784. doi: 10.2196/58784 (PMC12671306; doi:10.2196/58784)
Supplement: Multimedia Appendix 1 [file xr-v2-e58784-s001.docx]

Appendix 1. *SCRIBE checklist*

| Item number | Topic | Item description | Note |
| --- | --- | --- | --- |
| TITLE and ABSTRACT | | | |
| 1 | Title | Identify the research as a single-case experimental design in the title | pg. 1 |
| 2 | Abstract | Summarise the research question, population, design, methods including intervention/s (independent variable/s) and target behaviour/s and any other outcome/s (dependent variable/s), results, and conclusions | pg. 2 |
| INTRODUCTION | | | |
| 3 | Scientific background | Describe the scientific background to identify issue/s under analysis, current scientific knowledge, and gaps in that knowledge base | pgs. 4-5 |
| 4 | Aims | State the purpose/aims of the study, research question/s, and, if applicable, hypotheses | pg. 5 |
| METHODS | | | |
|  | DESIGN | | |
| 5 | Design | Identify the design (e.g., withdrawal/reversal, multiple-baseline, alternating-treatments, changing-criterion, some combination thereof, or adaptive design) and describe the phases and phase sequence (whether determined *a priori* or data-driven) and, if applicable, criteria for phase change | pgs. 6 |
| 6 | Procedural changes | Describe any procedural changes that occurred during the course of the investigation after the start of the study | N/A |
| 7 | Replication | Describe any planned replication | N/A |
| 8 | Randomisation | State whether randomisation was used, and if so, describe the randomisation method and the elements of the study that were randomised | N/A |
| 9 | Blinding | State whether blinding/masking was used, and if so, describe who was blinded/masked | N/A |
|  | PARTICIPANT/S or UNIT/S | | |
| 10 | Selection criteria | State the inclusion and exclusion criteria, if applicable, and the method of recruitment | pgs. 6-7 |
| 11 | Participant characteristics | For each participant, describe the demographic characteristics and clinical (or other) features relevant to the research question, such that anonymity is ensured | pgs. 11, table 2 |
|  | CONTEXT | | |
| 12 | Setting | Describe characteristics of the setting and location where the study was conducted | pg. 7 |
|  | APPROVALS | | |
| 13 | Ethics | State whether ethics approval was obtained and indicate if and how informed consent and/or assent were obtained | pg. 6 |
|  | MEASURES and MATERIALS | | |
| 14 | Measures | Operationally define all target behaviours and outcome measures, describe reliability and validity, state how they were selected, and how and when they were measured | pgs. 8-9, table 1 |
| 15 | Equipment | Clearly describe any equipment and/or materials (e.g., technological aids, biofeedback, computer programs, intervention manuals or other material resources) used to measure target behaviour/s and other outcome/s or deliver the interventions | pgs. 7-8 |
|  | INTERVENTIONS | | |
| 16 | Intervention | Describe intervention and control conditions in each phase, including how and when they were actually administered, with as much detail as possible to facilitate attempts at replication | pg. 7 |
| 17 | Procedural fidelity | Describe how procedural fidelity was evaluated in each phase | pg. 9, table 1 |
|  | ANALYSIS | | |
| 18 | Analyses | Describe and justify all methods used to analyse data | pg. 10 |
| RESULTS | | | |
| 19 | Sequence completed | For each participant, report the sequence actually completed, including the number of trials for each session for each case. For participant/s who did not complete, state when they stopped and the reasons | pg. 11 |
| 20 | Outcomes and estimation | For each participant, report results, including raw data, for each target behaviour and other outcome/s | pgs. 11-18, tables 3-4, figures 1-4 |
| 21 | Adverse events | State whether or not any adverse events occurred for any participant and the phase in which they occurred | pg. 11 |
| DISCUSSION | | | |
| 22 | Interpretation | Summarise findings and interpret the results in the context of current evidence | pgs. 19-21 |
| 23 | Limitations | Discuss limitations, addressing sources of potential bias and imprecision | pg. 21 |
| 24 | Applicability | Discuss applicability and implications of the study findings | pgs. 21-22 |
| DOCUMENTATION | | | |
| 25 | Protocol | If available, state where a study protocol can be accessed | N/A |
| 26 | Funding | Identify source/s of funding and other support; describe the role of funders | pgs. 6, 24 |
