# Supplementary material for: At-Home Virtual Reality Intervention for Patients With Chronic Musculoskeletal Pain: Single-Case Experimental Design Study
Source: JMIR XR Spat Comput. 2025 Mar 4;2:e58784. doi: 10.2196/58784 (PMC12671306; doi:10.2196/58784)
Supplement: Multimedia Appendix 3 [file xr-v2-e58784-s003.docx]

Appendix 3. *Topic list focus group*

**General experience**

How did you experience using the VR intervention?

What did you like about the VR intervention?

What did you not like about the VR intervention?

Which chapters of the VR intervention did you (not) play and why?

What effect did the VR intervention have on your chronic pain?

**Feasibility**

How did you like the way the VR intervention was offered?

What would be the best time to receive the VR intervention?

What would be the best duration for the VR intervention?

What did you think of the advised dosage of the VR intervention?

How did you like to use the VR intervention at home?

How did you like to use the VR intervention by yourself?

What did you think about the instruction on the VR intervention?

How can we improve the VR intervention and its administration?
