# Supplementary figures and images for: At-Home Virtual Reality Intervention for Patients With Chronic Musculoskeletal Pain: Single-Case Experimental Design Study
Source: JMIR XR Spat Comput. 2025 Mar 4;2:e58784. doi: 10.2196/58784 (PMC12671306; doi:10.2196/58784)

Appendix 4. *Visual analysis of diary data on physical and emotional functioning*


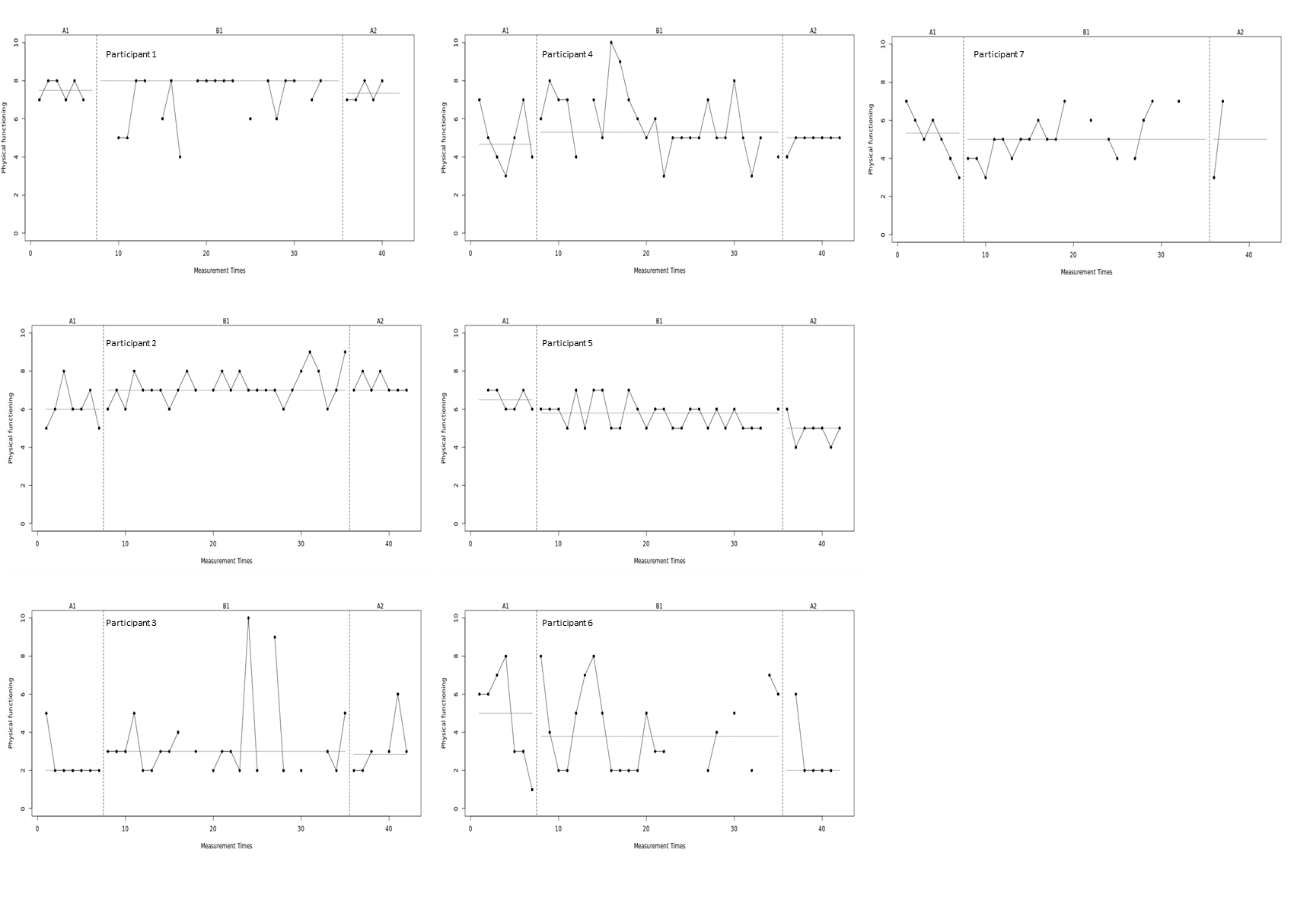


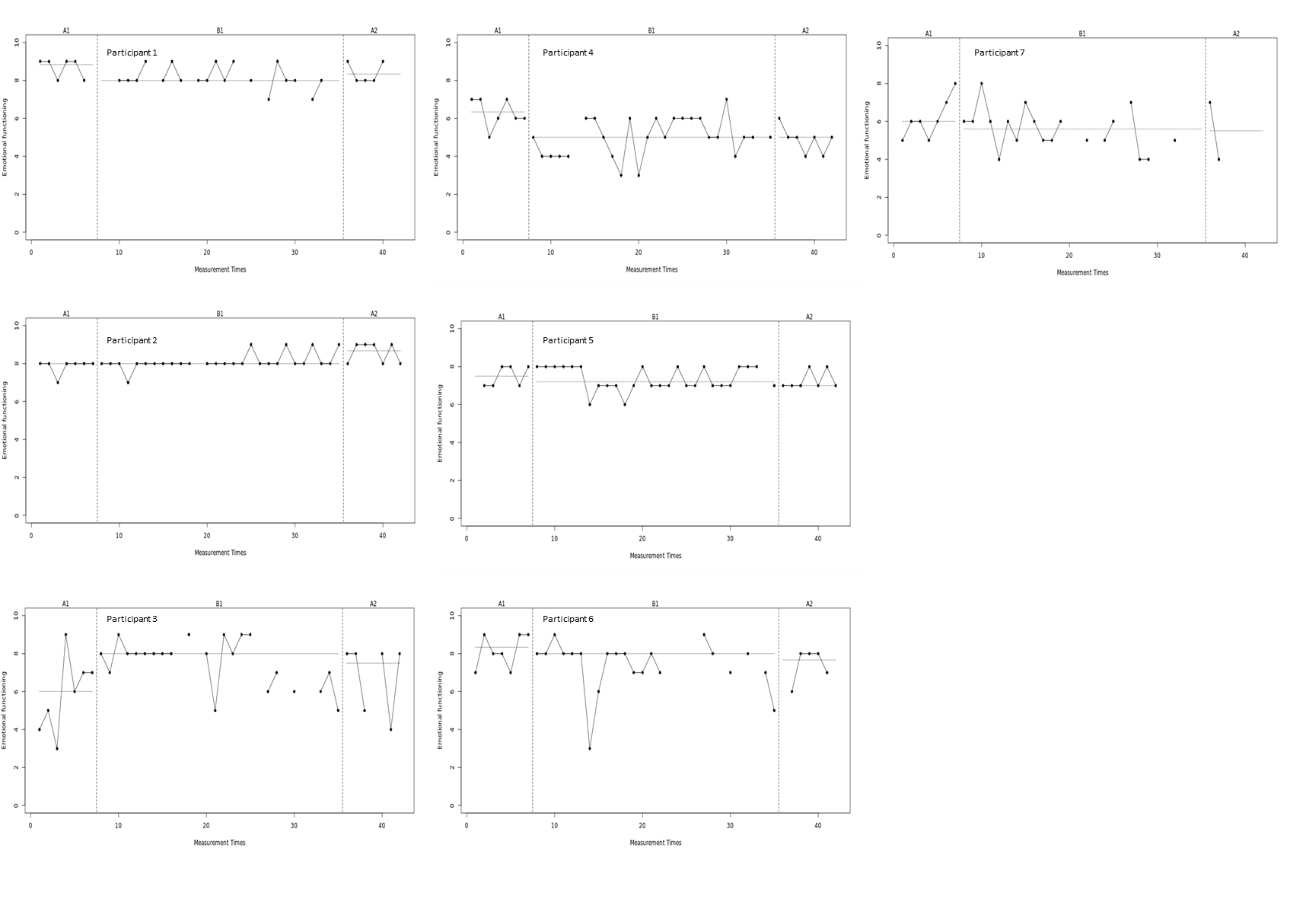

Supplement: Multimedia Appendix 4 [file xr-v2-e58784-s004.docx]

Appendix 5. *Individual scores on steps, stress and sleep*


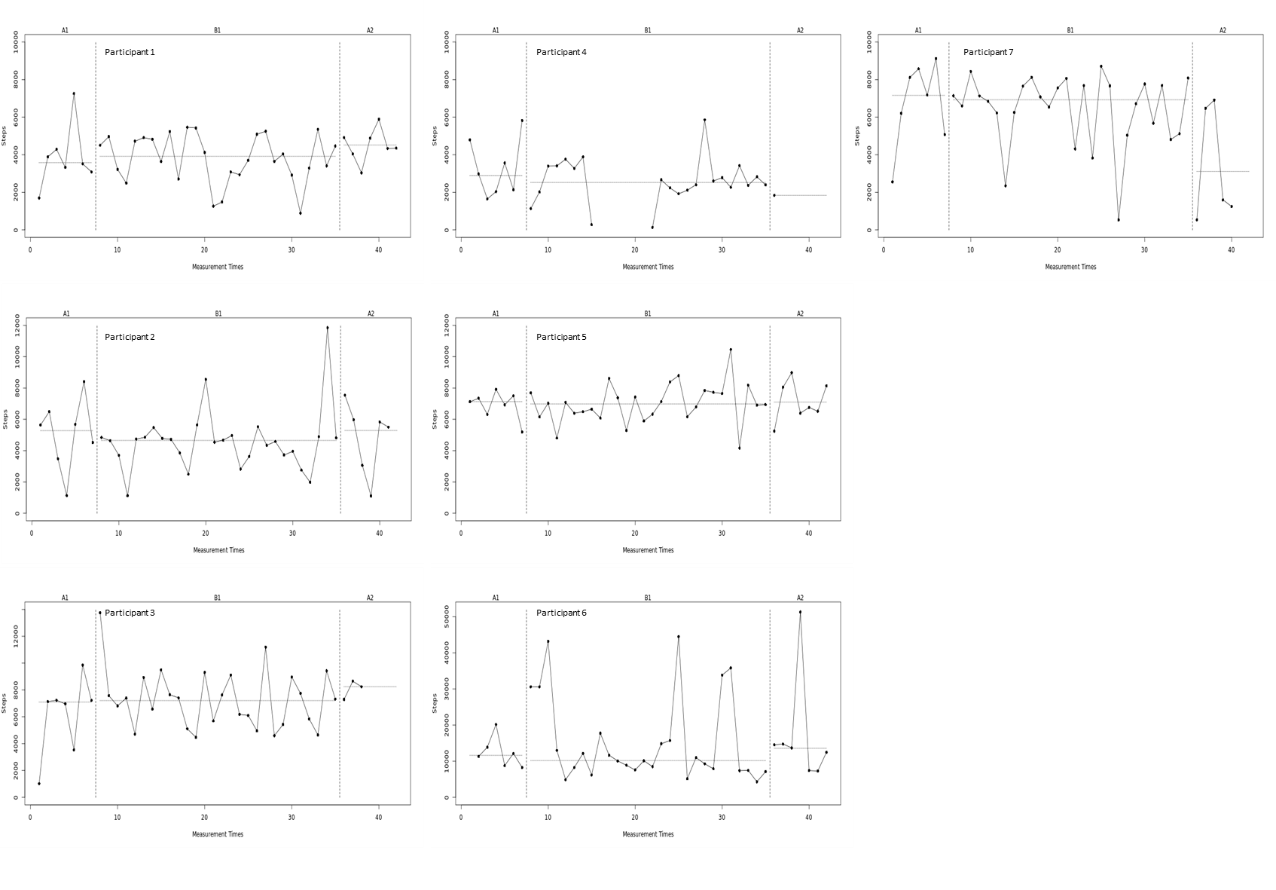


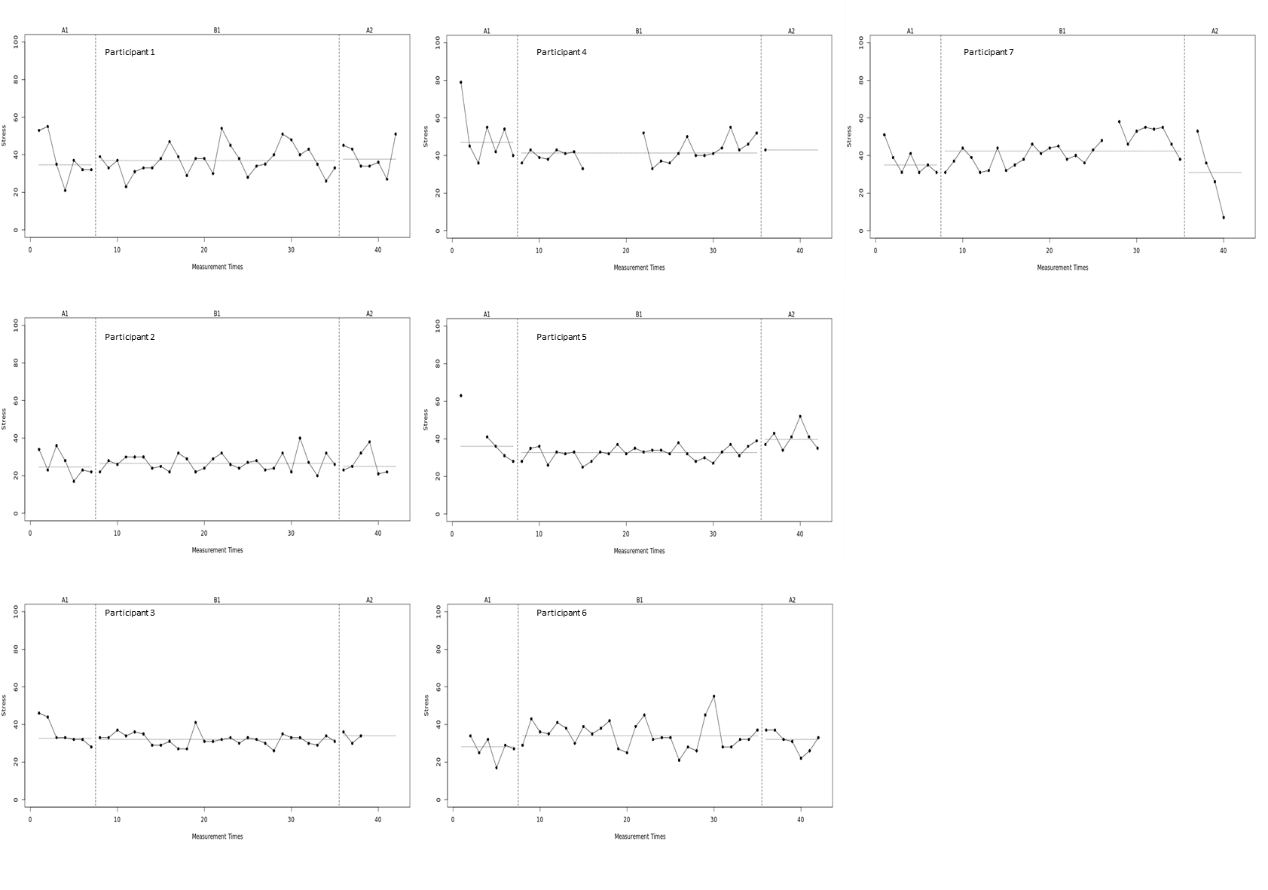


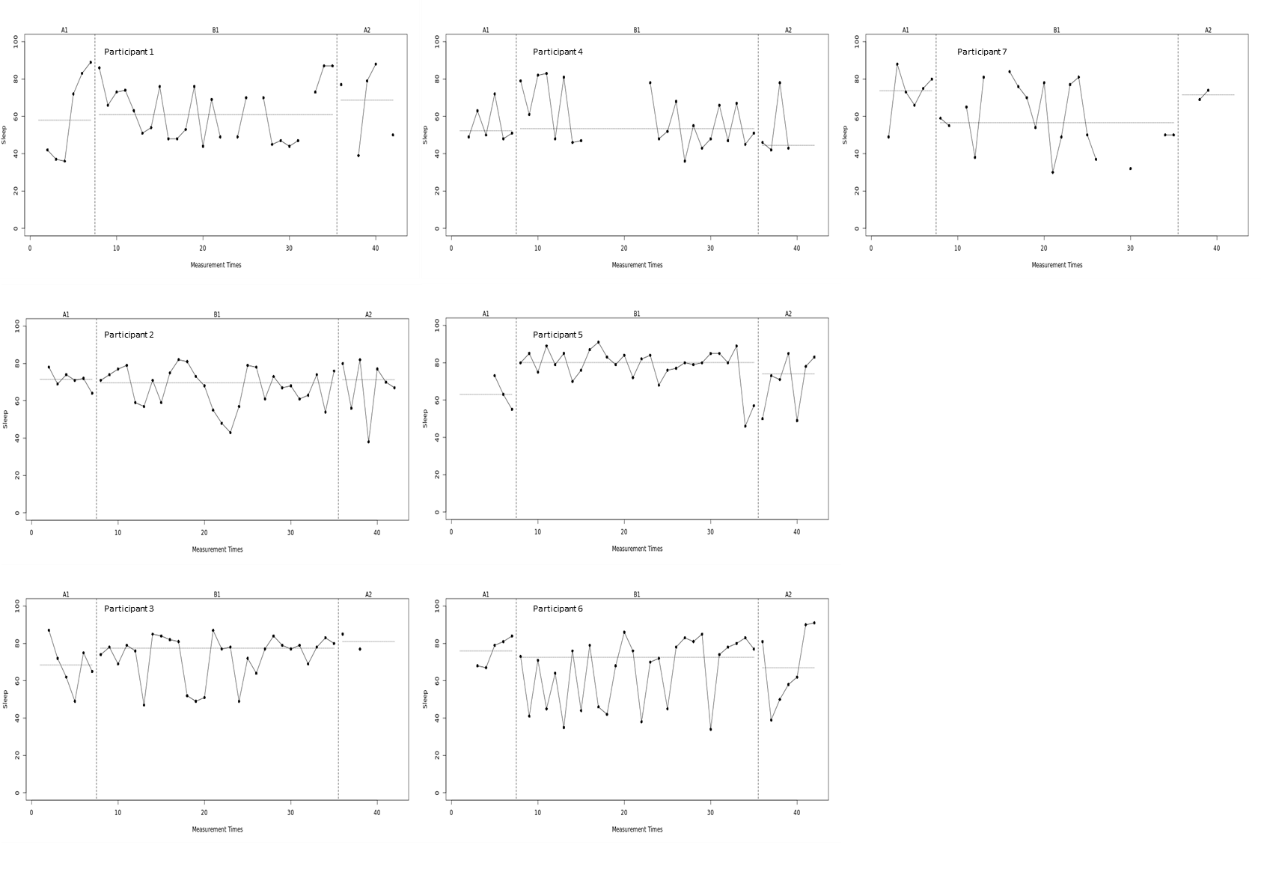

Supplement: Multimedia Appendix 7 [file xr-v2-e58784-s007.docx]
