# Supplementary material for: At-Home Virtual Reality Intervention for Patients With Chronic Musculoskeletal Pain: Single-Case Experimental Design Study
Source: JMIR XR Spat Comput. 2025 Mar 4;2:e58784. doi: 10.2196/58784 (PMC12671306; doi:10.2196/58784)
Supplement: Multimedia Appendix 5 [file xr-v2-e58784-s005.pptx]

## Slide 1
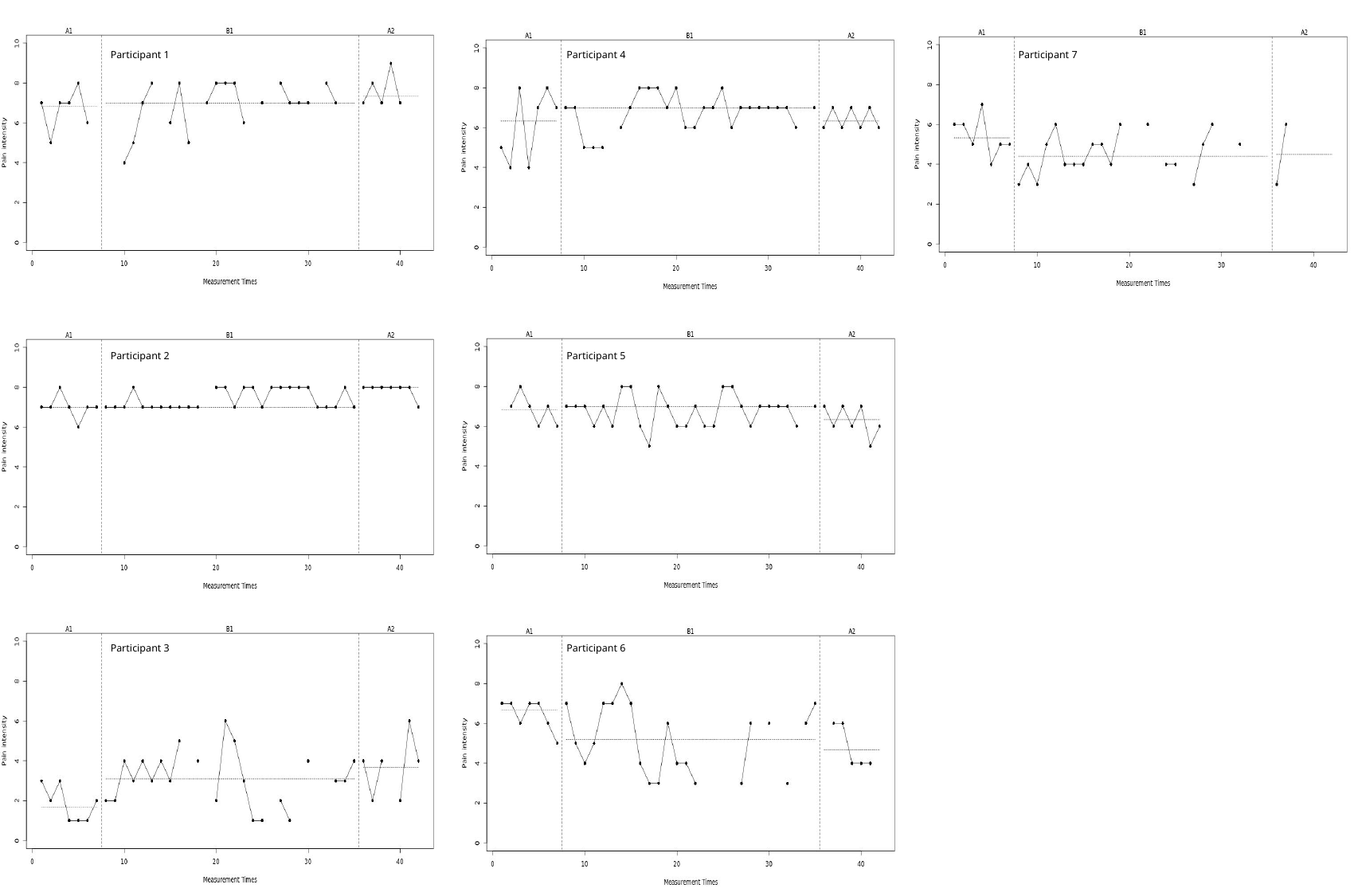

Participant 1
Participant 4
Participant 7
Participant 2
Participant 5
Participant 3
Participant 6
