# Supplementary material for: At-Home Virtual Reality Intervention for Patients With Chronic Musculoskeletal Pain: Single-Case Experimental Design Study
Source: JMIR XR Spat Comput. 2025 Mar 4;2:e58784. doi: 10.2196/58784 (PMC12671306; doi:10.2196/58784)
Supplement: Multimedia Appendix 8 [file xr-v2-e58784-s008.docx]

Appendix 6. *Group scores on weekly questionnaires*

|  | **1** | **2** | **3** | **4** | **5** | **6** | **7** |
| --- | --- | --- | --- | --- | --- | --- | --- |
| **PSEQ** |  |  |  |  |  |  |  |
| T0 | 43 | 28 | 45 | 15 | 33 | 21 | 27 |
| T1 | n/a | 33 | 39 | 27 | 40 | 25 | 27 |
| T2 | 42 | 33 | 44 | 29 | 36 | 19 | 27 |
| T3 | 46 | 31 | 46 | 26 | 37 | 23 | 30 |
| T4 | n/a | 39 | 45 | 26 | 40 | 16 | 24 |
| T5 | 36 | 36 | 45 | 23 | 44 | 19 | 29 |
| T6 | 40 | 36 | 48 | n/a | 46 | 16 | n/a |
| **PAQ** |  |  |  |  |  |  |  |
| T0 | 23 | 32 | 31 | 19 | 30 | 16 | 22 |
| T1 | n/a | 31 | 30 | 20 | 28 | 14 | 14 |
| T2 | 30 | 29 | 32 | 22 | 28 | 12 | 19 |
| T3 | 35 | 26 | 33 | 28 | 25 | 12 | 24 |
| T4 | n/a | 31 | 30 | 26 | 30 | 18 | 17 |
| T5 | 27 | 33 | 29 | 23 | 30 | 13 | 20 |
| T6 | 29 | 28 | 33 | n/a | 28 | 16 | n/a |
| **PCI Active** |  |  |  |  |  |  |  |
| T0 | 31 | 32 | 32 | 28 | 25 | 28 | 29 |
| T1 | n/a | 30 | 30 | 29 | 26 | 27 | 31 |
| T2 | 30 | 32 | 32 | 30 | 24 | 25 | 30 |
| T3 | 31 | 27 | 33 | 29 | 25 | 25 | 30 |
| T4 | n/a | 30 | 32 | 26 | 26 | 25 | 29 |
| T5 | 29 | 28 | 34 | 26 | 25 | 22 | 30 |
| T6 | 27 | 28 | 34 | n/a | 29 | 24 | n/a |
| **PCI Passive** |  |  |  |  |  |  |  |
| T0 | 40 | 40 | 42 | 66 | 43 | 48 | 53 |
| T1 | n/a | 48 | 42 | 62 | 48 | 49 | 47 |
| T2 | 38 | 48 | 41 | 60 | 41 | 50 | 56 |
| T3 | 41 | 49 | 39 | 61 | 42 | 43 | 57 |
| T4 | n/a | 47 | 36 | 66 | 47 | 44 | 59 |
| T5 | 46 | 44 | 35 | 59 | 43 | 45 | 55 |
| T6 | 40 | 43 | 36 | n/a | 44 | 45 | n/a |
